# Acetate promotes *SNAI1* expression by ACSS2-mediated histone acetylation under glucose limitation in renal cell carcinoma cell

**DOI:** 10.1042/BSR20200382

**Published:** 2020-06-15

**Authors:** Lv Yao, Linying Jiang, Fuxing Zhang, Minghua Li, Bo Yang, Fangting Zhang, Xiaoqiang Guo

**Affiliations:** 1 Department of Obstetrics and Gynecology, Sir Run Run Shaw Hospital, Zhejiang University School of Medicine, Hangzhou, Zhejiang 310016, China; 2 Central Laboratory, Peking University Shenzhen Hospital, Shenzhen 518036, China; 3 Guangdong and Shenzhen Key Laboratory of Male Reproductive Medicine and Genetics, Institute of Urology, Peking University Shenzhen Hospital, Shenzhen PKU-HKUST Medical Center, Shenzhen 518036, China; 4 Xiaobei Medical Research Institute, Department of Physical Education, Shijiazhuang Vocational College of Technology, Shijiazhuang, Hebei 050081, China

**Keywords:** Acetate, ACSS2, glucose limitation, histone acetylation, SNAI1

## Abstract

Metastasis is the main cause of cancer-associated deaths, yet this complex process is still not well understood. Many studies have shown that acetate is involved in cancer metastasis, but the molecular mechanisms remain to be elucidated. In the present study, we first measured the effect of acetate on zinc finger transcriptional repressor SNAI1 and acetyl-CoA synthetase 2 (ACSS2) under glucose limitation in renal cell carcinoma cell lines, 786-O and ACHN. Then, RNA interference and overexpression of ACSS2 were used to detect the role of acetate on SNAI1 expression and cell migration. Finally, chromatin immunoprecipitation assay (ChIP) was used to investigate the regulatory mechanism of acetate on SNAI1 expression. The results showed that acetate increased the expressions of SNAI1 and ACSS2 under glucose limitation. ACSS2 knockdown significantly decreased acetate-induced SNAI1 expression and cell migration, whereas overexpression of ACSS2 increased SNAI1 level and histone H3K27 acetylation (H3K27ac). ChIP results revealed that acetate increased H3K27ac levels in regulatory region of *SNAI1*, but did not increase ACSS2-binding ability. Our study identified a novel inducer, acetate, which can promote SNAI1 expression by ACSS2-mediated histone acetylation in partly. This finding has important implication in treatment of metastatic cancers.

## Introduction

There are many essential hallmarks of cancer, including uncontrolled cell survival, overgrowth, angiogenesis and metastasis [1]. Metastasis is the leading cause of cancer deaths [2], and approximately 90% of cancer patients die from metastasis. Unfortunately, the current understanding of metastasis is relatively limited.

Epithelial–mesenchymal transition (EMT) is a process by which epithelial cells increase their abilities of motility and invasiveness during embryonic development and organogenesis [3]. EMT has also been shown to play a critical role in promoting cancer metastasis [4]. Therefore, exploring the EMT mechanism is important for understanding cancer metastasis and finding preventive or therapeutic strategies [5]. Loss of E-cadherin is considered to be a fundamental event in EMT, which involves many transcription factors [6].

Zinc finger protein SNAI1 is a transcriptional repressor and also one of central mediators of EMT, which down-regulates the expression of E-cadherin [7]. Abnormal expression of SNAI1 exists in many metastatic cancers [8], which can be regulated by internal and external factors. Hypoxia can activate the expression of SNAI1 by hypoxia-inducible factor-1α [9]. Many types of histone modifications play a crucial role in the epigenetic regulation of SNAI1, including histone acetylation and methylation [10]. Histone H3K79 methyltransferase DOT1L can cooperates with histone acetyltransferase p300 to activate SNAI1 expression [11]. Another study has shown that histone H3K27 demethylase UTX is also involved in the epigenetic regulation of SNAI1 [12]. JMJD3 is another histone H3K27 demethylase and also up-regulates the expression of SNAI1 in breast cancer and gliomas [13,14]. Whether there are other inducible or epigenetic factors that regulate SNA1I needs further investigation.

Recently, it has been found that acetyl-CoA synthetase 2 (ACSS2) is related to the metastasis of renal cell carcinoma (RCC) [15,16]. In addition, ACSS2 is involved in hypoxia signaling and histone acetylation [17]. Based on these facts, we speculate that there may be a causal relationship between ACSS2 and SNAI1 expression.

In the present study, we found that acetate induced the expressions of SNAI1 and ACSS2 under glucose limitation in RCC cells. Knockdown of ACSS2 could inhibit acetate-induced SNAI1 expression and cell migration. Overexpression of ACSS2 increased SNAI1 level and histone H3K27 acetylation. Our results also indicated that acetate can increase histone acetylation in regulatory region of *SNAI1*, but cannot increase ACSS2-binding ability. The study reveals a new inducer of SNAI1 expression in epigenetic mechanism, which might become an important target for therapy of metastatic RCC.

## Materials and methods

### Cell lines and culture

Human renal cell adenocarcinoma cell lines 786-O and ACHN were purchased from cell resource center of Shanghai Institutes for Biological Sciences, Chinese Academy of Science. Both cells were cultured in DMEM (GIBCO, Grand Island, U.S.A.) or low glucose DMEM (4.5 mM glucose) supplemented with 10% heat-inactivated fetal bovine serum (FBS, Hyclone, Logan, U.S.A.), 100 U/ml penicillin and 100 mg /ml streptomycin (GIBCO) at 37°C in a 5% CO_2_, 95% air atmosphere. Acetate was purchased from Sangon (Shanghai, China).

### RNA interference for ACSS2

786-O and ACHN cells were seeded into six-well plates for 24 h and transfected with human ACSS2 siRNA and control siRNA using Lipofectamine 3000 (Invitrogen, Carlsbad, CA, U.S.A.) according to the manufacturer’s protocol. The efficiency of RNA interference (RNAi) was evaluated with qPCR and Western blotting at 48 h after transfection. Human ACSS2 siRNA and control siRNA were purchased from Genepharma (Shanghai, China). The knockdown sequences used for ACSS2 were 5′-CAGGAUUGAUGACAUGCUCAA-3′, and negative control sequences were 5′-UUCUCCGAACGUGUCACGU-3′.

### ACSS2 overexpression

The plasmids of pcDNA 3.1 and pcDNA 3.1-Flag-ACSS2 were provided as a generous gift by Dr Zhimin Lu at the University of Texas MD Anderson Cancer Center (Houston, TX, U.S.A.). Both 786-O and ACHN cells were plated in six-well plated and approximately 80%. The cells were transfected with plasmids using Lipofectamine 3000. The overexpressed efficiency was detected by Western blotting and qPCR at 48h after transfection.

### Scratch assay

A scratch assay was completed to determine the cell motile ability of both 786-O and ACHN cells. Briefly, cells were seeded on the six-well plates (Corning, NY, U.S.A.) and treated with RNAi and overexpression. At 6 h post-transfection, a clean line was created with a sterile pipette tip. The migration of cells was monitored using a digital camera system and imaged at the time of 0 and 24 h (for ACHN cells) or 30 h (for 786-O cells). The relative cell migration was quantified by dividing the migration width at specific time by the total width at the starting time (0 h).

### Quantitative polymerase chain reaction (qPCR)

RNA was extracted from cells using Trizol reagent (Invitrogen) and then reversely transcribed into cDNA with RT reagent Kit (TaKaRa, Dalian, China) according to the manufacturer’s protocol. qPCR analysis was carried out by LightCycler480 System (Roche, Foster City, CA, U.S.A.). β-Actin was used as an internal control to calculate the relative expression. The primers used in the study were synthesized by Sangon (Shanghai, China). The primer was designed in primerbank website (https://pga.mgh.harvard.edu/primerbank/). Sequences of the primer pairs used were as follows: *ACSS2* (5′-AAAGGAGCAACTACCAACATCTG-3′,5′-GCTGAACTGACACACTTGGAC-3′); *SNAI1* (5′-AGATGAGCATTGGCAGCGAG-3′, 5′-TCGGAAGCCTAACTA CAGCGA-3′); *β-ACTIN* (5′-CCACTGGCATCGTGATGGACTCC-3′, 5′-GCCGTG GTGGTGAAGCTG TAGC-3′).

### Western blotting

The cells were washed with cold PBS and were then collected using the scraper. The cells were lysed using lysis buffer (radioimmuno-precipitation assay, RIPA) containing the protease inhibitors cocktail for 30 min on ice. After centrifugation at 10,600 ***g*** at at 4°C for 15 min, the supernatants were collected. Fifty micrograms of total protein were loaded and separated by 10% sodium dodecyl sulfate-polyacrylamide gel electrophoresis (SDS-PAGE) and transferred to polyvinylidene difluoride (PVDF) membranes. The membranes were saturated with 5% skim milk in TBST (50 mM Tris–HCl, 150 mM NaCl, 0.1% Tween-20) and then incubated with primary antibodies at 4°C overnight. The primary antibodies used in the present study included rabbit polyclonal antibodies to ACSS2 (Sigma-Aldrich, St Louis, U.S.A.), SNAI1 (Cell Signaling Technology), acetyl H3K27 (Abcam, Shanghai, China), Histone H3 (CST) and β-Actin (Abcam). The membranes were incubated with HRP-conjugated goat anti-rabbit antibody (Cell Signaling Technology, Danvers, MA, U.S.A.) for 2 h at room temperature and then exposed to enhanced chemiluminescence substrate (Millipore, Rockford, U.S.A.), and detection was performed using a film. The quantification of Western blot is completed as follows. First, the relative value of specific protein was calculated by dividing its gray value with internal control (β-ACTIN or H3) gray value. Second, the final value of specific protein was obtained by dividing it relative value in the experimental group by in the control group (the final value in the control group was 1.00). The same method was used in other Figures. Western blotting results are representative of three independent experiments.

### ChIP-qPCR assays

Chromatin Immunoprecipitation (ChIP) was performed using EZ-ChIP kit (No 17-371, Upstate, Millipore, U.S.A.) according to the manufacturer’s protocol. ACHN cells were fixed in 1% (w/v) formaldehyde for 10 min at room temperature and fixation was quenched with the addition of glycine to 125 mM for a further 5 min. Cells were washed with cold 1× PBS for two times and lysed in SDS lysis buffer containing 1× Protease Inhibitor Cocktail II. Chromatin DNA was sonicated with 4–5 sets of 10-s pulses on ice and sheared to a length between 200 and 1000 bp using the JY92-II Ultrasonic Cell Crasher (Ningbo, China). The supernatant was collected by centrifugation at 12,000 ***g*** at 4°C for 10 min and pre-cleared with protein G agarose for 1 h at 4°C with rotation. Ten microliters of supernatant was saved as input. Chromatin was then incubated overnight with 1 μg RNA polymerase antibody (positive control), or 1 μg mouse IgG (negative control), or 3 μg ACSS2 antibody or 3 μg H3K27ac antibody per sample at 4°C with rotation. Protein G agarose was then added and incubated for a further 1 h at 4°C with rotation. The protein/DNA complexes were eluted at room temperature for 15 min. The DNA–protein cross-links were reversed by adding NaCl (final concentration 0.2 M) and then incubating at 65°C for 6 h. DNA was purified using spin columns. Finally, qPCR was completed to determine immunoprecipitation DNA content. The ChIP-enriched DNA samples were quantified by qPCR, and the data are expressed as a percentage of input. The primers used in SNAI1 ChIP were listed as follows: primer1 (5′-GGCACGGCCTAGCGAGT-3′, 5′-AGTGGTCGAGGCACTGGG-3′); primer2 (5′-AGCCCAGGCAGCTATTTCA G-3′, 5′-CTGGGAGACACATCGGTCAG-3′). The primer was designed with Primer3 tool (http://bioinfo.ut.ee/primer3-0.4.0/).

### Statistical analyses

Experimental values are shown as means ± standard deviation (SD) from at least three independent experiments. Statistical significance between two groups was determined using the paired two-tailed Student’s *t*-test. Two-way ANOVA was used for the comparison of more than two groups. *P* values less than 0.05 were considered to be statistically significant.

## Results

### Acetate increases SNAI1 and ACSS2 expressions under glucose limitation in RCC cells

**Figure 1:**
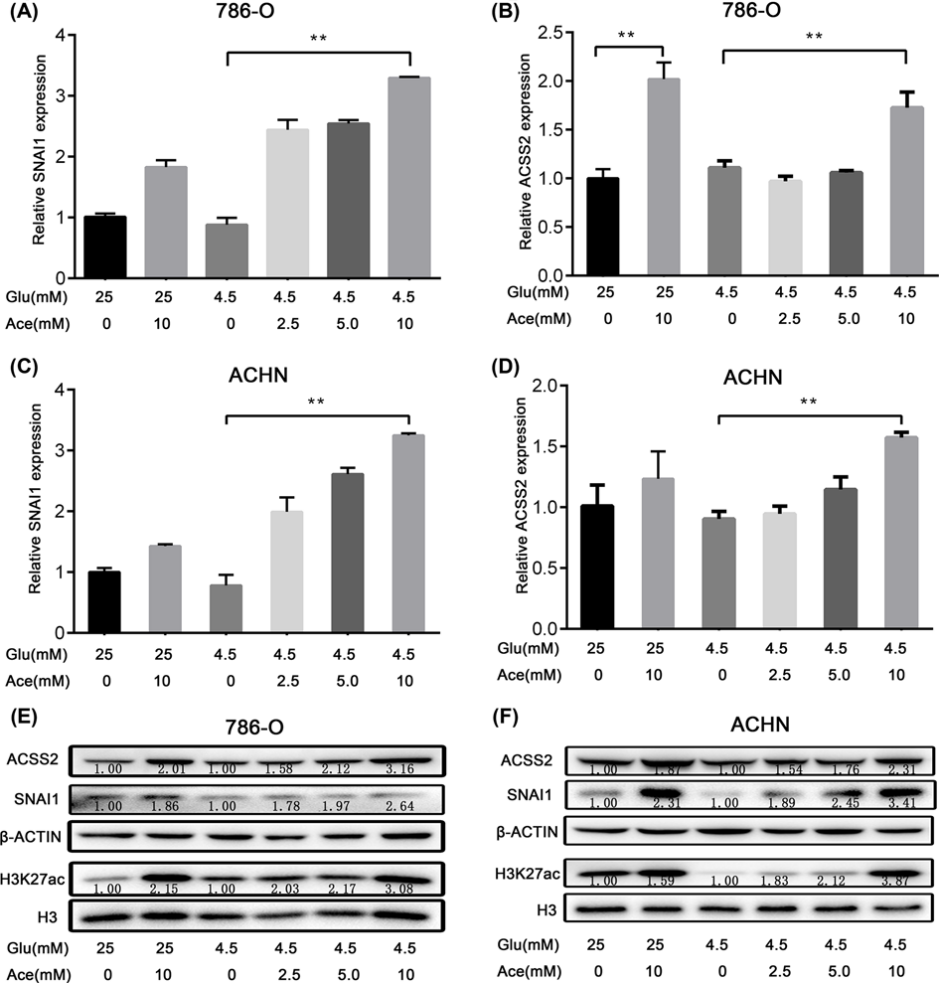
Acetate increased SNAI1 expression and histone acetylation The relative mRNA levels of SNAI1 with acetate supplement under standard glucose concentration (25 mM Glu) or glucose limitation (4.5 mM Glu) in 786-O (**A**) or ACHN cells (**C**). The relative mRNA levels of ACSS2 with acetate supplement under standard glucose concentration or glucose limitation in 786-O (**B**) or ACHN cells (**D**). The protein content of SNAI1, ACSS2 and acetylated H3K27 (H3K27ac) under standard glucose concentration or glucose limitation in 786-O (**E**) or ACHN cells (**F**). (*, *P*<0.05; **, *P*<0.01; Two-way ANOVA)

Dysregulated metabolism is a hallmark of cancer. Cancer cells have to use a lot of energy materials other than glucose for rapid proliferation, such as lactate and acetate. Previous studies have shown that acetate participates in many biological processes and regulates the expression of specific genes, such as erythropoietin (EPO) and fatty acid synthase (FASN) [18–20]. In our study, we first measured the regulatory role of acetate on SNAI1 expression in kidney cancer cell lines 786-O and ACHN. The results showed that acetate could significantly increase the mRNA and protein contents of SNAI1 at 10 mM under glucose limitation (4.5 mM) (Figure 1A,C,E,F). However, the regulatory role of acetate is not obvious under standard glucose content (25 mM).

Acetate can be converted to acetyl CoA, which is catalyzed by acetyl-CoA synthetase (ACSS), including ACSS 1 and ACSS 2 [21]. ACSS 1 is located in mitochondrion and mainly involved in ATP production [22]. ACSS2 is found not only in the cytoplasm to support lipid synthesis, but also in the nucleus for histone acetylation [23,24]. Since the process of gene expression regulation is mainly accomplished in the nucleus, we focus on the effects of acetate on ACSS2 and histone acetylation. We found that acetate supplement up-regulated the ACSS2 expression at mRNA and protein levels (Figure 1B,D,F), and also increased H3K27 acetylation under glucose limitation (Figure 1E,F). Previous studies have proved that acetate regulates histone acetylation by specifically inducing nuclear localization of ACSS2 during oxygen and serum limitation [20], and our results showed that acetate also affected histone acetylation by increasing ACSS2 expression under glucose limitation. Collectively, these data imply that acetate is implicated in the regulation of SNAI1 under glucose limitation and is also associated with ACSS2 and histone acetylation.

### Knockdown of ACSS2 inhibited acetate-induced SNAI1 up-regulation and cell invasion under glucose limitation

**Figure 2:**
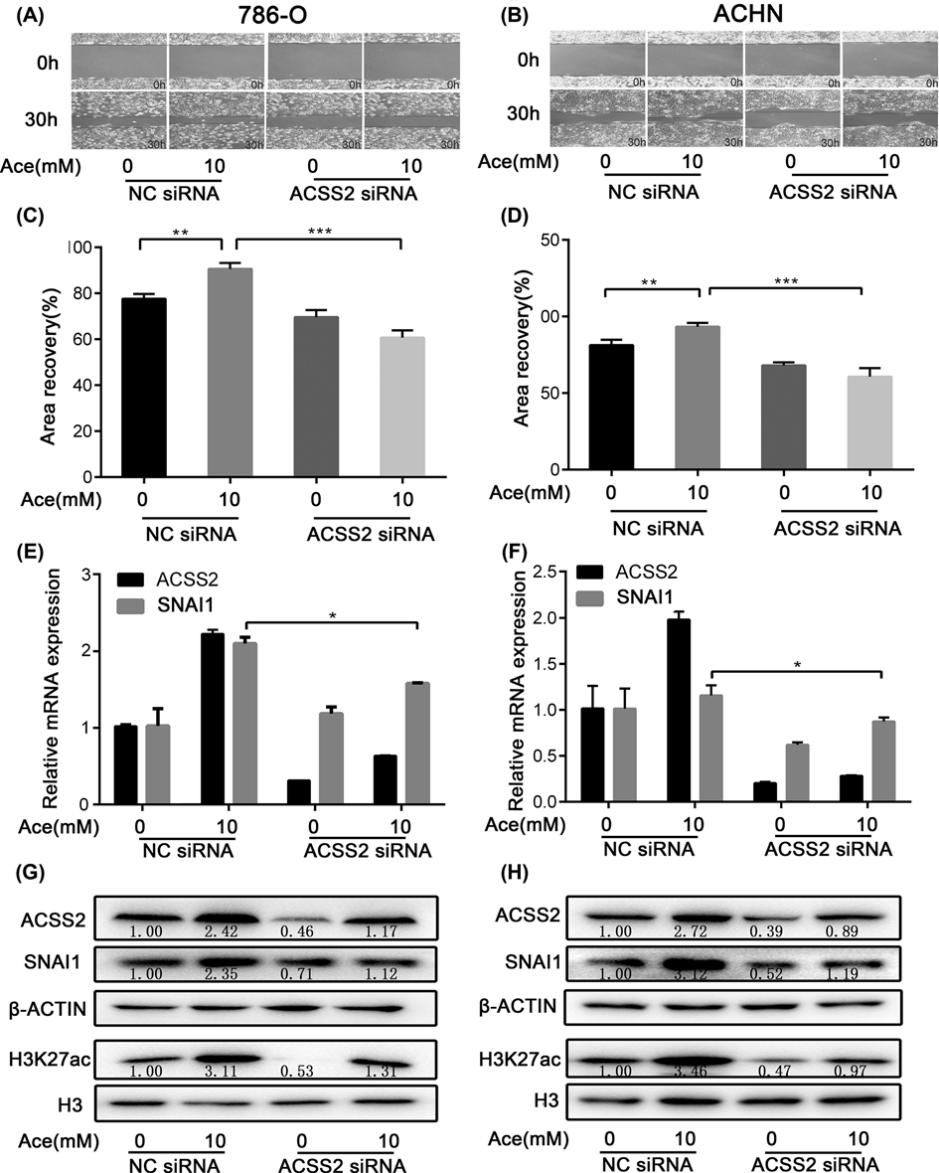
ACSS2 knockdown inhibited acetate-induced SNAI1 expression and cell migration under glucose limitation (**A**) The difference of cell migration ability in non-specific control 786-O cells (NC siRNA) and ACSS2 knockdown cells (ACSS2 siRNA). (**B**) The difference of cell migration ability in control ACHN cells (NC) and ACSS2 knockdown cells. (**C**) Quantitative results of 786-O cell migration. (**D**) Quantitative results of ACHN cell migration. (**E**) The relative mRNA levels of ACSS2 and SNAI1 in control 786-O cells (NC) and ACSS2 knockdown cells. (**F**) The relative mRNA levels of ACSS2 and SNAI1 in control ACHN cells (NC) and ACSS2 knockdown cells. (**G**) The protein content of ACSS2, SNAI1 and H3K27ac in control 786-O cells (NC) and ACSS2 knockdown cells. (**H**) The protein content of ACSS2, SNAI1 and H3K27ac in control ACHN cells (NC) and ACSS2 knockdown cells (*, *P*<0.05; **, *P*<0.01; ***, *P*<0.001 two-tailed Student’s *t*-test).

We and others have demonstrated that ACSS2 can promote metastasis of RCC [15,16], but the underling mechanism remains to be investigated. To determine the role of ACSS2 in acetate-induced SNAI1 expression, we performed ACSS2 knockdown experiments under glucose limitation. The data showed that RNA interference of ACSS2 significantly inhibited the expression of SNAI1 induced by acetate, and also reduced H3K27 acetylation level (Figure 2E–H). Previous studies have shown that acetate is an important inducer of cancer metastasis [25,26]. Consistently, we also confirmed that the knockdown of ASCC2 significantly blocked the motility of RCC cells (Figure 2A–D), which further demonstrated that ACSS2 played an important role in acetate-induced metastasis.

### ACSS2 overexpression promoted the expression of SNAI1 and cell invasion

**Figure 3:**
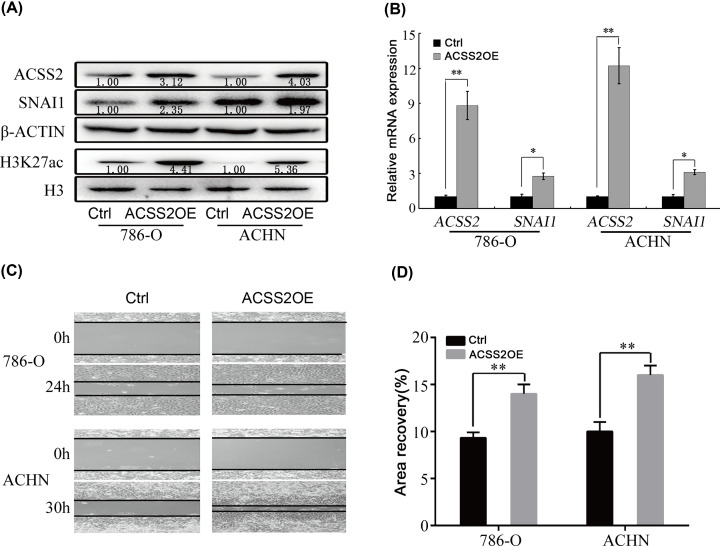
Ectopic overexpression of ACSS2 promoted SNAI1 expression and cell migration (**A**) The protein content of ACSS2, SNAI1 and H3K27ac in control cells (Ctrl) and ACSS2-overexpressed cells (ACSS2OE). (**B**) The relative mRNA levels of ACSS2 and SNAI1 in Ctrl cells and ACSS2OE cells. (**C**) The difference of cell migration ability in Ctrl cells and ACSS2OE cells. (**D**) Quantitative results of cell migration. (*, *P*<0.05; **, *P*<0.01; two-tailed Student’s *t*-test).

The overexpression effect of ACSS2 on SNAI1 was further investigated under glucose limitation. The data indicated that overexpression of ACSS2 significantly up-regulated SNAI1 expression and increased H3K27 acetylation (Figure 3A,B). In addition, overexpression of ACSS2 also obviously promoted cell migration of RCC cells (Figure 3C,D). These results further suggested that ACSS2 is an important mediator of acetate-induced SNAI1 expression and cell migration.

### ACSS2 increased SNAI1 expression by promoting histone acetylation in regulatory regions of SNAI1

**Figure 4:**
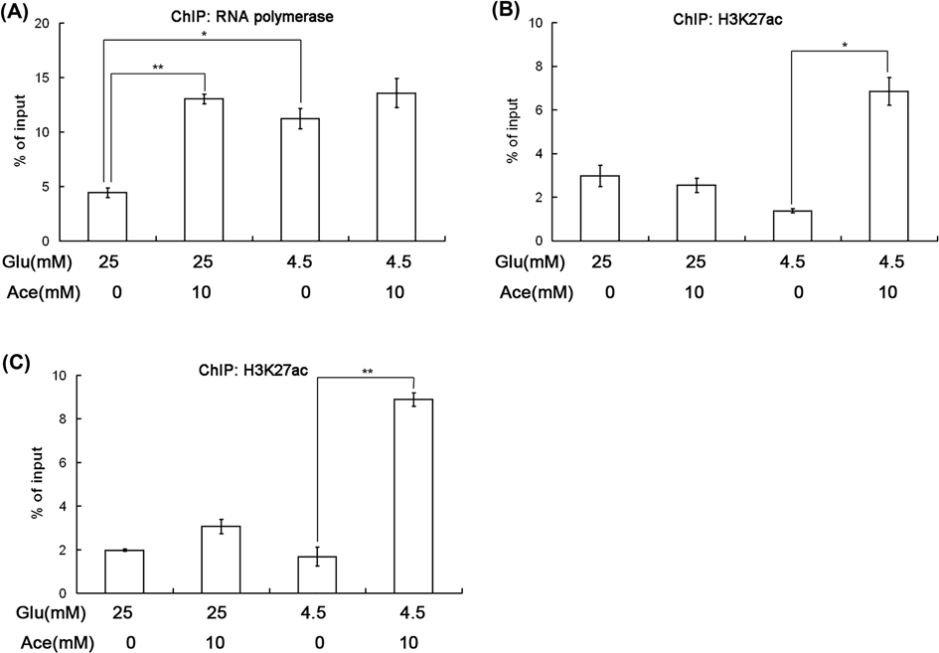
Histone acetylation promoted acetate-induced SNAI1 expression ChIP analysis of H3K27ac on the regulatory regions of *SNAI1* (**A**). The binding of RNA polymerase Ⅱ to fragment 1 of regulatory regions of *SNAI1*. (**B**) The H3K27 acetylation enrichment at fragment 1 of regulatory regions of *SNAI1*. (**C**) The H3K27 acetylation enrichment at fragment 2 of regulatory regions of *SNAI1*. (*, *P*<0.05; **, *P*<0.01; two-tailed Student's *t*-test).

It is well known that histone acetylation is one of the basic characteristics of transcriptional activation [27]. To explore the potential mechanism of SNAI1 expression induced by acetate, we performed ChIP-qPCR assays and measured the histone acetylation in regulatory regions of SNAI1 under glucose limitation. The results showed that acetate increased transcriptional and H3K27 acetylation level by acetate supplementation in regulatory regions of SNAI1 (Figure 4A–C). However, the binding of ACSS2 to regulatory regions of SNAI1 did not increased significantly after acetate addition (data not shown). Collectively, these data demonstrated that acetate promoted SNAI1 expression through increasing histone acetylation.

## Discussion

Alteration in the metabolism facilitates cancer development and progression [28]. To overcome unfavorable conditions such as glucose deficiency and hypoxia, cancer cell increases the utilization of alternative fuels, including amino acids, ketone bodies and acetate [29]. Acetate is particularly important because it can produce acetyl-CoA, which not only provides carbon source for cancer cell biomass accumulation but also acts as an epigenetic regulator of histone acetylation [30]. Our study provides further evidence that acetate and its derivative, acetyl-CoA, play an important role in expression of SNAI1 and cancer cell migration.

Aberration in metabolic enzymes is also important for cancer development [31]. As an acetate-utilizing enzyme located in nucleus, ACSS2 catalyzes the production of acetyl CoA which plays an important role in histone acetylation and gene expression regulation [32]. Previous study has shown that there is a link between acetyl-CoA generation ‘on-site’ at chromatin for histone acetylation and the transcription of specific genes [33]. Another study also showed that acetyl-CoA impacts H3K27ac and gene expression at specific loci [34]. In the present study, our results clearly indicate that the acetyl-CoA catalyzed by ACSS2 is a key factor in the regulation of SNAI1 expression.

Previous study has shown that ACSS2 promoted RCC cell migration and invasion through activating PI3K/AKT signaling pathway [14]. Our previous study found that ACSS2 can promote cell invasion of RCC by up-regulating lysosomal-associated membrane protein 1 expression [15]. The present study revealed a new mechanism of ACSS2 involved in cell invasion, that is, activation of SNAI1 by histone acetylation under glucose limitation.

Recent studies have demonstrated that metabolic reprogramming plays a key role during cancer progression [35]. Specific dietary nutrients can affect cancer metabolic reprogramming [36]. Acetate, produced by alcohol metabolism or from microbiota [37,38], can promote cancer progression by metabolic reprogramming and histone acetylation. Therefore, the present study is of great significance to explain the mechanism of cancer metastasis and develop new treatment strategies.

**Figure 5:**
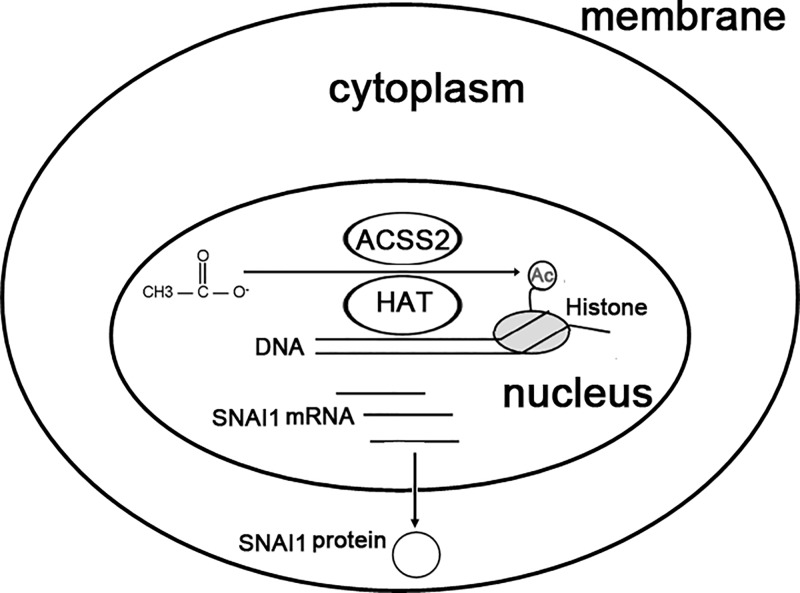
A mechanism of acetate-induced SNAI1 expression Glucose limitation increases acetate uptake and utilization. Acetate can be catalyzed into acetyl-CoA in nucleus by ACSS2 for H3K27 acetylation, which is important for *SNAI1* transcriptional activation; Ac, acetyl group; HAT, histone acetyltransferase.

Alterations in histone modification and abnormalities of gene expression regulation play important role in metastasis of RCC. It is found that there is increased H3K27 acetylation in RCC [39]. Previous studies have also shown that high SNAI1 expression indicates poor survival of RCC patients [40]. Based on the above analysis, we propose a work model that acetate induces SNAI1 expression under glucose limitation (Figure 5).

## Conclusion

The present study revealed that acetate is an important inducer of SNAI1 expression in RCC, suggesting that acetate may also promote the metastasis of RCC. Our results also provided evidence that acetate promotes SNAI1 expression through ACSS2-mediated histone acetylation, implying that inhibition of ACSS2 may be an important strategy in the treatment of metastatic tumors.

## Abbreviations

ACSS2: acetyl-CoA synthetase 2
ChIP: chromatin immunoprecipitation
DMEM: Dulbecco’s modified Eagle’s medium
EMT: epithelial–mesenchymal transition
FBS: fetal bovine serum
H3K27: histone H3 Lys27
HAT: histone acetyltransferase
qPCR: quantitative polymerase chain reaction
RCC: renal cell carcinoma
RNAi: RNA interference
